# Bone conduction facilitates self-other voice discrimination

**DOI:** 10.1098/rsos.221561

**Published:** 2023-02-15

**Authors:** Pavo Orepic, Oliver Alan Kannape, Nathan Faivre, Olaf Blanke

**Affiliations:** ^1^ Laboratory of Cognitive Neuroscience, Neuro-X Institute and Brain Mind Institute, Faculty of Life Sciences, École polytechnique fédérale de Lausanne (EPFL), 1202 Geneva, Switzerland; ^2^ Virtual Medicine Centre, NeuroCentre, University Hospital of Geneva, 1205 Geneva, Switzerland; ^3^ University Grenoble Alpes, University Savoie Mont Blanc, CNRS, LPNC, 38000 Grenoble, France; ^4^ Department of Clinical Neurosciences, University Hospital of Geneva, 1205 Geneva, Switzerland

**Keywords:** self-voice, self-other voice discrimination, bone conduction, familiar voice, multi-sensory integration, self-other voice space

## Abstract

One's own voice is one of the most important and most frequently heard voices. Although it is the sound we associate most with ourselves, it is perceived as strange when played back in a recording. One of the main reasons is the lack of bone conduction that is inevitably present when hearing one's own voice while speaking. The resulting discrepancy between experimental and natural self-voice stimuli has significantly impeded self-voice research, rendering it one of the least investigated aspects of self-consciousness. Accordingly, factors that contribute to self-voice perception remain largely unknown. In a series of three studies, we rectified this ecological discrepancy by augmenting experimental self-voice stimuli with bone-conducted vibrotactile stimulation that is present during natural self-voice perception. Combining voice morphing with psychophysics, we demonstrate that specifically self-other but not familiar-other voice discrimination improved for stimuli presented using bone as compared with air conduction. Furthermore, our data outline independent contributions of familiarity and acoustic processing to separating the own from another's voice: although vocal differences increased general voice discrimination, self-voices were more confused with familiar than unfamiliar voices, regardless of their acoustic similarity. Collectively, our findings show that concomitant vibrotactile stimulation improves auditory self-identification, thereby portraying self-voice as a fundamentally multi-modal construct.

## 1. Introduction

We are all familiar with the strange sensation that occurs when we hear our voice in video or voice recordings [1–5]. Considering the fundamental role our voice plays in our everyday communication, this should be quite surprising. We have a lifelong daily exposure to our voice, higher than exposure even to the most familiar voices. Our own voice is the sound most intimately linked to our self. Although there is ample evidence showing that self-related stimuli are perceived differently and activate distinct cortical regions compared with other, non-self-associated stimuli [6–14], the specific mechanisms of self-voice perception have been surprisingly under-investigated, both in behavioural and neuroimaging studies [15–17]. For instance, the extent to which self-voice perception differs from that of other familiar voices remains poorly understood; as does the extent to which acoustic properties that enable discriminating voices of other people [18] are involved in self-other voice discrimination (VD). A better understanding of self-voice perception is of immediate clinical relevance, as deficits in self-other VD have been related to auditory-verbal hallucinations (AVHs) [19–22] (i.e. ‘hearing voices’), one of the most common [23,24] and most distressing [25,26] hallucinations in a major psychiatric disorder, schizophrenia. Investigating different perceptual factors underlying self-other VD, we here hypothesized that one key contribution would stem from bone conduction and, based on our findings, propose a new experimental paradigm that improves the ecological validity for studying self-voice perception.

A crucial contribution for the perception of our own voice, and our own voice only, comes from bone conduction resulting from speech production/articulation. Under natural conditions, one's spoken voice is transmitted not only through the air, but also, unfailingly through the skull [27,28], which alters self-voice perception in two ways. First, due to the different sound propagation, bone conduction transforms the sound of our voice—specifically, it is assumed to instantiate a low-pass filter [29,30]. Because of the low-frequency emphasis, we hear our voice as lower [29] compared with how our voice sounds to others. Second, next to transforming the sound of our voice, bone conduction conveys additional sensory information, as not only auditory, but also vibrotactile [31] and somatosensory [32,33] signals are involved, resulting from the vibrations of the skull and skin deformation. Thus, self-voice, when heard under natural conditions, is not only an auditory but rather a multi-modal percept.

One reason for the scarcity of self-voice studies probably lies in methodological obstacles faced when creating appropriate experimental stimuli. Without bone conduction, prior self-voice studies inevitably contain a perceptual mismatch between the experimental self-voice stimuli (e.g. presented through air-conducting loudspeakers) and the actual self-voice. In fact, the majority of studies that compared recognition of self-voice versus other voices reported lower accuracy rates and higher response times for self-voice compared with other voices [16,34–48]. Early self-voice studies suggested that this discrepancy between self- and other voices might result from a lower previous exposure to self-voice in voice recordings [34,35,37]. However, similar behavioural differences still persist [16,36–41,45], with a higher exposure to recorded self-voice through contemporary technology (e.g. voice messages and video recordings). Moreover, more recent self-voice paradigms often demonstrate ceiling effects [37,39–41,46–49], e.g. high accuracy rates in all experimental conditions, reflecting a need for more sensitive experimental paradigms. To account for the aforementioned ecological discrepancy, several studies investigated if acoustic transformations (e.g. low-pass or other types of filters) of air-conducted self-voice stimuli would render the self-voice more natural to the listeners. These attempts, however, yielded contradictory results [50–54], as they indicated preferences for different acoustic transformations. Crucially, these studies manipulated only one aspect related to bone conduction effects on self-voice (i.e. acoustic transformations) and neglected the additional vibrotactile stimulation. In order to better approximate natural self-voice, experimental self-voice stimuli should be accompanied with the concomitant vibrotactile stimulation resulting from the vibrations of the skull. Here, we address this by providing vibrational input through a bone conduction headset and investigate whether it improves self-voice perception, as opposed to auditory input alone.

In a series of three behavioural studies in independent cohorts, and using a new self-voice perception paradigm, we investigated the following three main perceptual factors of self-other VD: (i) sound conduction type (air versus bone), (ii) other-voice familiarity (familiar versus unfamiliar), and (iii) acoustic voice parameters. Using voice-morphing technology [55] and bone conduction headphones, we designed a psychophysical self-other VD task to investigate the nature of perceptual differences in self-other VD, while trying to avoid ceiling effects. Participants heard short voice morphs of their own and other people's vocalizations (phoneme /a/) and indicated whether the morphs more closely resembled their own or someone else's voice. In Study 1 (*N* = 16), we investigated differences in self-other VD as a function of sound conduction (air, bone) and how this is modulated by previous exposure to self-voice [34,35,37]; in Study 2 (*N* = 16), we extended this to familiar-other VD in order to investigate whether the bone conduction effects are specific for self-voice, or generalize to other familiar voices [56,57]. In Study 3, we set out to replicate Studies 1 and 2 within a single, larger cohort (*N* = 52). We, furthermore, included an additional self-familiar VD task and a control self-voice recognition task (without voice morphing) and investigated the acoustic parameters of all tested voices [18]. We hypothesized that bone conduction would facilitate self-voice perception in self-other VD (bias or increased sensitivity) (Study 1) but would not affect familiar-other VD task (Study 2). We further hypothesized that bone conduction effects would be more prominent without exposure to the self-voice used in our experiment prior to the task—i.e. when the task difficulty is increased (Studies 1 and 2)—and that they would occur regardless of other-voice familiarity [56,57] (Study 3).

## 2. Methods

### 2.1. Participants

Studies 1 and 2 each involved 16 participants. In Study 1, seven participants were male (mean age ± s.d.: 29.7 ± 5.5 years old) whereas eight were male in Study 2 (28.5 ± 5.5 years old). For Study 3, participants were accompanied by an acquaintance (a friend) of the same gender and similar age, who also participated in the study, and it involved 52 participants (20 male, 26.5 ± 4.6 years old). Nine out of 52 participants were excluded from the main regression analysis in Study 3, based on their low performance in the control task (see Procedure). In Study 3, we recruited participants in pairs as this allowed participants to provide both self- and familiar voices (see Procedure). All participants were right-handed, reported no hearing deficits, and no history of psychiatric or neurological disorders. They were chosen from the general population and were naive to the purpose of the study. Participants gave informed consent in accordance with institutional guidelines (protocol 2015-00092, approved by the Comité Cantonal d'Ethique de la Recherche of Geneva) and the Declaration of Helsinki, and received monetary compensation (CHF 20 h^−1^).

The sample size for Study 3 was selected based on power analysis of Study 1, which indicated that a sample size of *n* = 47 provides greater than 84% power (95% CI = [75.32, 90.57]) for the interaction between Conduction and Voice Morph with the effect size of −0.12 (100 simulations, α = 0.05).

### 2.2. Procedure

#### 2.2.1. Study 1: self-other voice discrimination

**Figure 1.**
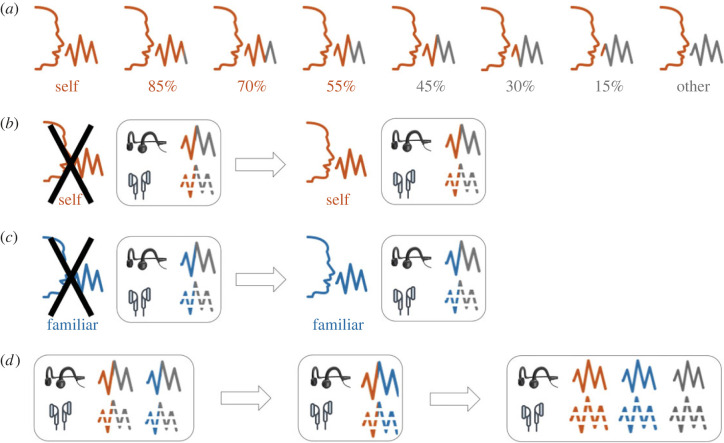
Experimental design of Studies 1–3. Colours represent different types of voices: self (orange), unfamiliar other (grey) and familiar other (blue). Blocks represent different types of auditory tasks (self-other, familiar-other, self-familiar, and control). In all studies, all tasks were performed with bone (solid line) and with air (dashed line) conduction (black and white headphone icons, respectively), separated in experimental blocks. (*a*) Experimental stimuli for the self-other task in Studies 1 and 3. Six voice morphs were sampled from self-other voice continuum generated with voice-morphing technology. Equivalent voice morphs were used in other discrimination tasks (familiar-other and self-familiar). (*b*) Study 1 design. Two blocks (with bone and air conduction) of self-other task were first performed without (self-voice icon crossed out) and then with self-voice shown prior to the task (previous exposure to self-voice). (*c*) Study 2 design. Two blocks (with bone and air conduction) of familiar-other task were first performed without (familiar-voice icon crossed out) and then with familiar-voice shown prior to the task (previous exposure to familiar voice). (*d*) Study 3 design. Self-voice and familiar voice were first discriminated against the unfamiliar voice and then against each other. The control task in which self-voice was detected among the three unmorphed voices was conducted at the end of Study 3.

In Study 1, we morphed each participant's voice with the voice of a gender-matched unfamiliar person. For each voice morph, participants were instructed to indicate whether the voice they heard more closely resembled their own or someone else's voice by pressing on one of two buttons. Based on our previous work [58,59], six voice ratios (% self-voice: 15, 30, 45, 55, 70, 85; figure 1*a*) were chosen and repeated 10 times within a block in a randomized order (total of 60 trials). The study contained four experimental blocks, which differed based on the sound conduction type (air, bone) and whether participants were exposed to the unmorphed self-voice immediately prior to the experiment. In the first two blocks, participants performed the task without having previously heard the unmorphed recording of their voice, once with each type of sound conduction, whereas before the remaining two blocks the unmorphed self-voice was presented to participants (figure 1*b*). The order of air- and bone-conduction blocks was counterbalanced across participants and for both parts of the experiment (with and without previous exposure to self-voice).

#### 2.2.2. Study 2: familiar-other voice discrimination

In Study 2, the experimental design (figure 1*c*) was equivalent to Study 1, except that the self-voice was substituted with the voice of a familiar other. Should the effects in Study 1 be caused by the familiarity with one's own voice and not one's own voice *per se* (i.e. the other voice was not familiar), then we would expect similar performance in Study 2. Thus, participants heard voice morphs between a familiar other voice and an unfamiliar other voice, either via air or via bone conduction, and either without (first two blocks) or with previous exposure to a familiar voice. In each trial, they indicated whether the corresponding morph more closely resembled the familiar voice or someone else's.

#### 2.2.3. Study 3: self-other, familiar-other, and self-familiar voice discrimination

In Study 3, we contrasted self-other and familiar-other VD tasks in the same, independent cohort of participants. Moreover, in the same group of participants, we performed a self-familiar VD task, thereby investigating whether the bone conduction effects persist irrespective of other-voice familiarity. Thus, Study 3 consisted of two parts (figure 1*d*). In the first part, participants performed two blocks of the self-other VD task (air or bone, cf. Study 1) and two blocks of the familiar-other task (air or bone, cf. Study 2), using the counterbalanced order. This was followed by two blocks of self-familiar VD task (air or bone) that were counterbalanced across participants. Self-familiar blocks were always conducted after self-other and familiar-other blocks to balance the exposure to self- and to familiar voice for their discrimination from the unfamiliar voice, before they were tested against each other.

#### 2.2.4. Study 3: control self-voice recognition task

At the end of Study 3 (figure 1*d*), participants performed a control self-voice recognition task in which, unbeknown to participants, the stimuli consisted only of unmorphed voices (self, familiar, and unfamiliar), and, as opposed to the VD tasks, all three voices were used as stimuli within the same experimental block. In each trial, participants were instructed to indicate whether the voice they hear resembled their voice by pressing a button. There were two control task blocks, one for each form of sound conduction (air or bone), counterbalanced across participants. Each of the three unmorphed voices was randomly repeated 10 times within the block. As this task served as a control to identify whether participants were able to recognize their unmorphed recorded voice, it was always performed at the end of experiment, so as not to affect the performance in the discrimination tasks by previous exposure to unmorphed voice recordings.

### 2.3. Stimuli and materials

Prior to participating in the studies, participants' voices were recorded while vocalizing the phoneme /a/ for approximately 1 to 2 s (Zoom H6 Handy recorder). Each recording was normalized for average intensity (−12 dBFS) and duration (500 ms) and cleaned from background noise (Audacity software). In detail, immediately before recording participants’ voices, we recorded a few seconds of baseline background noise, that was filtered out from the vocalization. The 500 ms clips that were used as stimuli were selected from the utterance by avoiding its onset and offset, and additionally by selecting its most stable part (i.e. 500 ms that do not noticeably vary in sound intensity). Noise reduction parameters in Audacity software were set to default (12 dB, sensitivity: 6, smoothing: 3 bands). Cleaning of background noise did not significantly alter the formants of the voice stimuli. The distance between the recorder and participants was not controlled for, but it was always around 20 cm, and the sound intensity was normalized for each recording, rendering them standardized across participants. In principle, participants vocalized /a/ only once, unless the recording was not of good quality (e.g. too short so that either onset or offset could not be avoided when selecting the 500 ms interval, or varying noticeably in sound intensity across the 1–2 s of the recording).

In Studies 1–3, such preprocessed voice recordings were used to generate voice morphs spanning a voice identity continuum between two voices by using TANDEM-STRAIGHT [55] (e.g. a voice morph can be generated such that it contains 40% of person A's and 60% of person B's voice).

In Study 1, the other voice was a voice of a gender-matched unfamiliar person. In Study 2, the familiar voice belonged to a male person with whom participants were acquainted. In Study 3, participants came with a gender-matched acquaintance who also participated in the study and whose voice served as familiar-other voice. The gender-matched unfamiliar voices were the same in all studies.

In Studies 1 and 2, the unmorphed voices in blocks with previous exposure were presented to participants through the same sound conduction type used for that experimental block (air or bone).

In Study 3, as air-conduction medium, we used headphones (Bose QC20) instead of laptop loudspeakers (GIGABYTE AORUS x5, Studies 1 and 2). Both air- and bone-conducting headphones were installed on participants' heads before the beginning of the experiment and matched for loudness at lower sound intensities, such that vibrotactile sensations resulting from bone conduction could not be perceived, resulting in participants being unable to determine the source of the auditory stimuli throughout the experiment. This served as a stricter methodology, as it enables a better concordance in sound intensity and spatial location between the bone- and air-conducted stimuli. However, we did not formally quantify the extent to which the participants were able to determine the source of the auditory stimuli—this was only inferred from participants’ reports. Despite this difference in air-conduction medium, i.e. loudspeakers (Study 1) versus headphones (Study 3), we observed similar effects in comparison with bone conduction in both studies (see Results). In all studies, we used the same Aftershokz Sportz Titanium headphones as bone-conducting medium.

In all studies, inter-trial intervals jittered between 1 and 1.5 s to avoid predictability of stimulus onset.

All studies were performed in Matlab 2017b with Psychtoolbox library [60].

### 2.4. Statistical analysis

#### 2.4.1. Voice discrimination tasks

In all studies, the data were analysed with binomial mixed-effects regressions with Response as dependent variable, indicating whether participants perceived the presented voice morph as resembling their voice (self-other and self-familiar VD) or the familiar voice (familiar-other VD). In Studies 1 and 2, the regressions contained two fixed effects with an interaction term: Conduction (air, bone) and Previous Exposure (yes, no), as well as a fixed effect of Voice Morph (15, 30, 45, 55, 70, 85%). In Study 3, the effect of sound conduction on each type of VD (self-other, familiar-other, and self-familiar) was analysed with mixed-effects binomial regressions with Response as dependent variable and Conduction (air, bone) and Voice Morph (15, 30, 45, 55, 70, 85%), together with a two-way interaction, as fixed effects. For all mixed-effects regressions in all studies, random effects included a by-participant random intercept, and by-participant random slopes for the main effects were added following model selection based on maximum likelihood. Trials with reaction times greater or smaller than two interquartile ranges from the median for each participant were considered as outliers and excluded. Additionally, a linear mixed-effects regression with Reaction Times as a dependent variable and the same fixed and random effects was performed for all studies, with the polynomial expansion of the Voice Morph variable to level 2 (electronic supplementary material). To further validate null findings of the mixed-effects binomial regressions, we performed equivalent models relying on the Bayesian framework.

Statistical tests were performed with R [61], using the lme4 [62], lmerTest [63] and cocor [64] packages. The results were illustrated using sjplot [65] and ggplot2 [66] packages. Power analysis was performed with simr [67] package. Bayesian models were created in Stan computational framework (http://mc-stan.org/) accessed with the brms package [68].

#### 2.4.2. Control task and other-to-self-voice confusion

The performance in the control task of Study 3 was also analysed with mixed-effects binomial regressions with Response as dependent variable and two fixed effects with an interaction term: Conduction (air, bone) and Voice (self, familiar, unfamiliar).

For the control task of Study 3, we additionally explored whether self-voice was more misperceived as the familiar or with the unfamiliar voice. For that purpose, we correlated the rate of ‘other’ response in the self-voice trials (i.e. miss rate) with the rates of ‘self’ response in both familiar- and unfamiliar-voice trials (i.e. false-alarm (FA) rate). Pearson and Filon's *z*-test for comparing two correlations based on dependent groups with overlapping variables [69] was used to compare these two correlations (miss rate with two types of FA rates—familiar-as-self and unfamiliar-as-self misperception). The two FA rates were also correlated with each other. Where significant, separate correlations were then conducted for and compared between the two forms of sound conduction (air, bone; electronic supplementary material).

#### 2.4.3. Self-other voice discrimination acoustic analysis

We subsequently investigated whether the physical acoustic parameters that have been shown to account for the discrimination of other voices [18] also impact VD for one's own voice. Participants' unmorphed voices were placed in voice spaces as defined by Baumann & Belin [18], whose axes represent different acoustic parameters of the voices. In this space, similarly sounding voices are located close to each other and inter-voice distances have been correlated with other-voice discriminability [18] and related to the activity in auditory cortex [70]. A two-dimensional voice space was created [18,71,72], with the dimensions corresponding to contributions of source (pitch, larynx) and filter (formants, vocal tract) in voice production [73] (figure 4*a*). The voice spaces were normalized such that the origin of the spaces corresponds to the other voice in each self-other voice pair. The distance to the origin thus represents the acoustic difference between self- and other voices.

In detail, for each voice recording, we extracted the fundamental frequency (F0) and five formants (F1–F5) using Praat software [74] and computed its voice-space coordinates, corresponding to source (*x* coordinate) and filter (*y*) components of voice production [73] (males: *x* = log(F0), *y* = log(F5 – F4); females: *x* = log(F0), *y* = log(F1)). The choice of coordinates was based on the work by Bauman & Belin [18], who demonstrated that this combination of acoustic parameters best accounts for subjective discriminability of voices for each gender. As an exploratory analysis, we constructed several different voice spaces by using different acoustic parameters for the *y*-axis. The results remained unaltered and are placed in the electronic supplementary material. The coordinates were first transformed into *z*-scores, after which the voice spaces were normalized for the other voice, such that other-voice coordinates were subtracted from self-voice coordinates in each self-other voice pair. This resulted in a coordinate system where Euclidean distance to the origin represented self-other voice distance in *z*-score units. Z-scoring coordinates enabled us to place all participants (male and female) in the same voice space.

Distances to the origin (self-other voice distances) were then correlated with the percentage of correct responses in self-other VD task. In the same way, we created familiar-other voice space and compared familiar-other distances with familiar-other task performances. Significant correlations were run again for and compared between the two forms of sound conduction (air, bone; electronic supplementary material). Acoustic parameters of all participants’ voices are reported in the electronic supplementary material.

## 3. Results

### 3.1. Study 1: self-other voice discrimination

**Figure 2.**
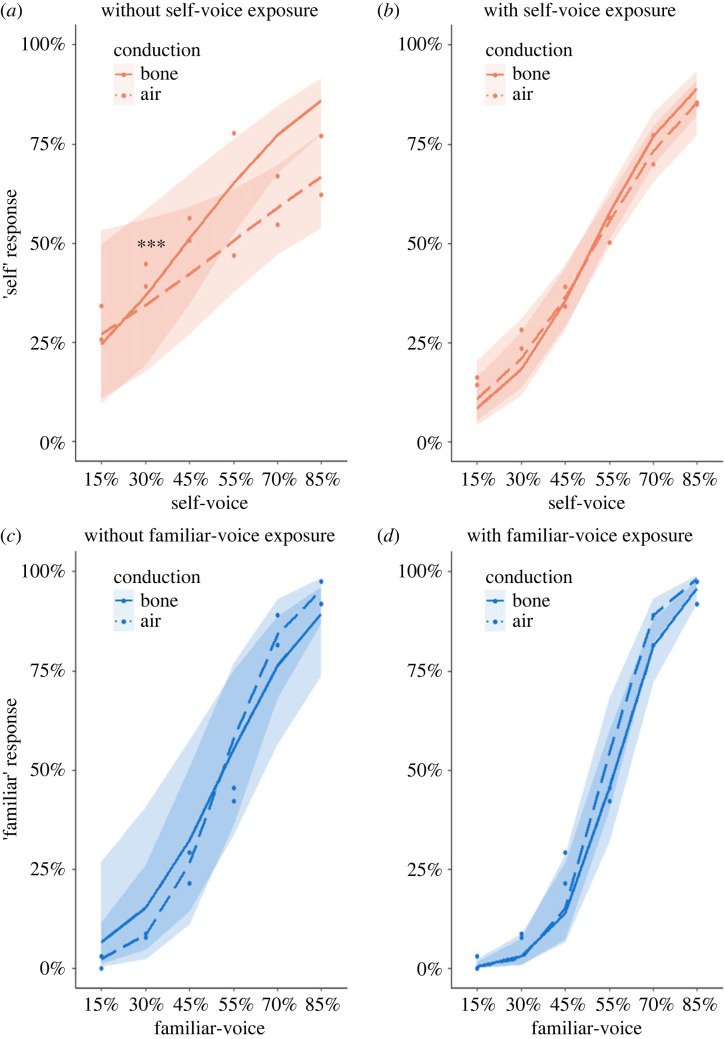
Studies 1 and 2. Psychometric curves fitted for two forms of sound conduction (bone—solid line; air—dashed) during studies 1 (self-other VD, (*a*) and (*b*)) and 2 (familiar-other VD, (*c*) and (*d*)). The abscissa indicates the percentage of the self/familiar voice present in a voice morph and the ordinate indicates the rate at which the corresponding voice morph was perceived as resembling the self/familiar voice. The shaded areas around each curve represent the 95% confidence intervals of the local estimates. Left plots ((*a*) and (*c*)) indicate perception for the blocks without and right plots ((*b*) and (*d*)) for the blocks with immediate previous exposure to the target voice prior to the task. Bone conduction improved self-unfamiliar discrimination only when participants were not previously exposed to their voice before the task (*a*). No such effects were observed for familiar–unfamiliar discrimination. *** *p* < 0.001.

In Study 1, participants discriminated between their voice and a voice of a gender-matched unfamiliar person, both through air conduction and through bone conduction. For each self-other voice morph (figure 1*a*), participants indicated whether the voice they heard more closely resembled their own or someone else's voice. In the first two blocks, participants performed the task without having previously heard the unmorphed recording of their voice, whereas before the remaining two blocks the unmorphed self-voice was presented to participants (figure 1*b*). As hypothesized, mixed-effects binomial regressions revealed main effects of Conduction (estimate = −0.47, *Z* = −2.96, *p* = 0.003), indicating a higher intercept of the psychometric curve fitted for bone conduction (figure 2), of Previous Exposure (estimate = −0.5, *Z* = −4.64, *p* < 0.001), indicating a higher intercept of the psychometric curve fitted for the blocks without previous exposure, and of Voice Morph (estimate = 0.55, *Z* = 22.67, *p* < 0.001), indicating that the ratio of ‘self’ response increased with increased amount of self-voice present in voice morphs (figure 2). Moreover, the analysis yielded a significant interaction between Conduction and Previous Exposure (estimate = 0.43, *Z* = 2.85, *p* = 0.004). In order to investigate the nature of the interaction, we ran a separate mixed-effects binomial regression for each type of Previous Exposure. The analysis for the blocks without previous exposure to self-voice revealed a significant interaction between Voice Morph and Conduction (estimate = −0.25, *Z* = −3.46, *p* < 0.001), indicating a steeper slope for the psychometric curve fitted for bone conduction, compared with the curve fitted for air conduction (figure 2*a*). On the contrary, for the blocks with previous exposure to self-voice, the effect of Conduction did not interact with the effect of Voice Morph (estimate = −0.12, *Z* = −1.63, *p* = 0.104) (figure 2*b*). The observed difference in slopes shows that participants performed the self-other VD task better when stimuli were presented through bone conduction, compared with air conduction, but only without previous exposure to unmorphed self-voice recordings. In both *post hoc* analyses, there was a main effect of Voice Morph (no previous exposure: estimate = 0.59, *Z* = 4.39, *p* < 0.001; previous exposure: estimate = 0.9, *Z* = 7.28, *p* < 0.001) but not of Conduction (no previous exposure: estimate = 0.39, *Z* = 1.38, *p* = 0.17; previous exposure: estimate = 0.4, *Z* = 1.4, *p* = 0.16).

Collectively, the results of Study 1 indicate that participants are better at discriminating self- and other voices (i) when voice morphs are presented through bone conduction, (ii) that previous exposure makes the self-other VD task easier, and (iii) that this bone conduction-related enhanced self-perception disappears when subjects are exposed before the task to their own unmorphed voice stimuli.

### 3.2. Study 2: familiar-other voice discrimination

The observed effect of enhanced perception of the self-voice in Study 1 may be caused by the effects of familiarity with one's own voice and not one's own voice *per se*. Hence, in Study 2, the experimental design (figure 1*c*) and statistical analysis were equivalent to Study 1, except that the self-voice was substituted with the voice of a familiar other.

As hypothesized, the mixed-effects binomial regression in the familiar-other discrimination with Response as dependent variable only revealed a significant effect of Voice Morph (estimate = 1.16, *Z* = 30.53, *p* < 0.001), indicating that the rate of ‘familiar’ response increased with increased amount of familiar voice present in voice morphs. Also, as hypothesized, the effects of Conduction (estimate = −0.03, *Z* = −0.22, *p* = 0.826) and Previous Exposure (estimate = −0.54, *Z* = −1.17, *p* = 0.242) were not significant, nor was their interaction (estimate = 0.27, *Z* = 1.41, *p* = 0.159). Equivalent models relying on the Bayesian framework revealed evidence in favour of the null hypothesis according to which sound conduction did not affect familiar-other discrimination (Bayes factor = 0.17, see electronic supplementary material).

These data show that familiar-other discrimination was not significantly affected by the type of sound conduction or previous exposure to the familiar-voice (figure 2*c,d*). Thus, they suggest that the effects observed in Study 1 involve self-related processes rather than those of familiarity.

### 3.3. Study 3: self-other, familiar-other, and self-familiar voice discrimination

Results of Studies 1 and 2 showed that the bone conduction effects are specific to self-voice and do not generalize to other familiar voices, respectively, albeit in different groups of participants. Hence, in the first part of Study 3, we contrasted self-other and familiar-other VD tasks in the same, independent cohort of participants (figure 1*d*). In addition, in the second part of Study 3, we performed a self-familiar VD task in the same group of participants, to investigate whether the observed bone conduction effects are dependent on other-voice familiarity (figure 1*d*).

**Figure 3.**
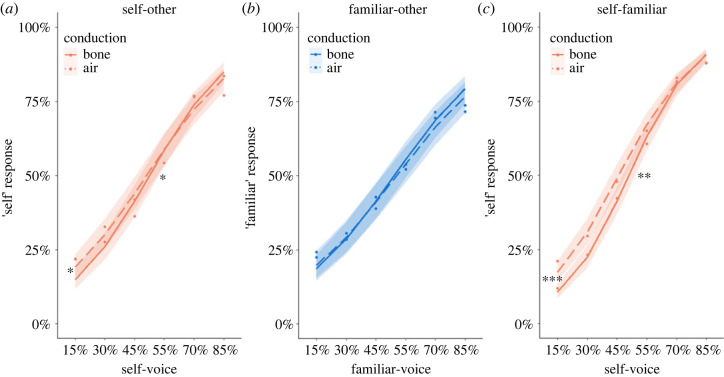
Study 3: voice discrimination tasks. Psychometric curves fitted for two forms of sound conduction (bone—solid line; air—dashed) during all three voice discrimination tasks ((*a*) self-other; (*b*) familiar-other; (*c*) self-familiar). The abscissa indicates the percentage of the self/familiar voice present in a voice morph and the ordinate indicates the rate at which the corresponding voice morph was perceived as more resembling the self/familiar voice. The shaded areas around each curve represent the 95% confidence intervals of the local estimates. Asterisks in the lower end of the curves indicate a significant difference in intercepts, whereas asterisks in the middle of the curves indicate a significant difference in slopes. Intercepts were lower and the slope was steeper for the curves fitted for bone conduction, but only in the self-related tasks ((*a*) and (*c*)). * *p* < 0.05, ** *p* < 0.01, *** *p* < 0.001.

The results of the three discrimination tasks (self-other, familiar-other, and self-familiar) are illustrated in figure 3. Replicating Studies 1 and 2, we observed a significant interaction between the effects of Conduction and Voice Morph, characterized by a steeper psychometric curve for bone compared with air conduction, but only in tasks involving self-voice (self-other, self-familiar) (self-other: estimate = −0.1, *Z* = −2.26, *p* = 0.024; familiar-other: estimate = −0.05, *Z* = −1.12, *p* = 0.263; self-familiar: estimate = −0.13, *Z* = −2.71, *p* = 0.007). Self-related tasks also had a significant effect of Conduction, showing a lower intercept for bone conduction (self-other: estimate = 0.4, *Z* = 2.44, *p* = 0.015; familiar-other: estimate = 0.12, *Z* = 0.75, *p* = 0.452; self-familiar: estimate = 0.7, *Z* = 3.97, *p* < 0.001). Equivalent models relying on the Bayesian framework revealed evidence in favour of the null hypothesis according to which sound conduction did not affect familiar-other discrimination (Bayes factor = 0.21, see electronic supplementary material). As before, all three tasks indicated a main effect of Voice Morph, indicating an increase in ‘self’/’familiar’ answers with an increase with the amount of self/familiar voice present in voice morphs (self-other: estimate = 0.7, *Z* = 22.13, *p* < 0.001; familiar-other: estimate = 0.56, *Z* = 19.09, *p* < 0.001; self-familiar: estimate = 0.89, *Z* = 25.15, *p* < 0.001).

Overall, these data demonstrate that bone conduction improved the performance in VD tasks if the task involved self-voice morphs, regardless of other-voice familiarity (steeper psychometric curves in self-other and self-familiar, but not in familiar-other task; asterisks in the middle of plots in figure 3). Lower intercepts for bone conduction (asterisks in the left end of plots in figure 3) indicate that this was especially prominent for other-dominant voice morphs (i.e. containing lower rate of self-voice present) [59].

### 3.4. Self-other voice discrimination acoustic analysis

We subsequently investigated whether the physical acoustic parameters that have been shown to account for the discrimination of other voices [18] also impact VD for one's own voice. Participants' unmorphed voices were placed in a self-other voice space in which similar voices are located close to each other and the distance to the origin represents the acoustic difference between self- and other voices.

**Figure 4.**
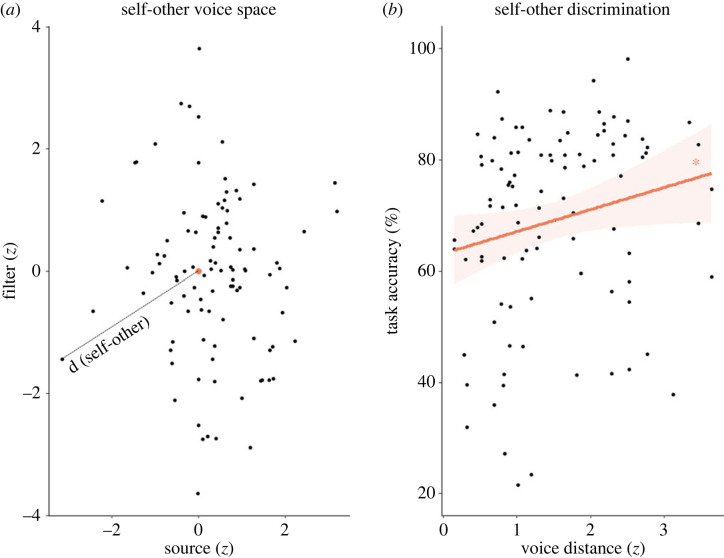
Self-other voice discrimination—acoustic analysis. (*a*) Voice space in which the origin (enlarged orange dot) represents the other voice in self-other discrimination tasks. Distance to the origin (dashed line) thus represents each participant's self-other voice distance in *z*-score units. (*b*) Self-other distances were correlated to the self-other task performances. Shaded area around linear regression indicates 95% confidence interval. * *p* < 0.05.

Correlation analysis indicated a positive association between self-other voice distances and self-other task performance (both for self-familiar and self-unfamiliar tasks) (*r* = 0.2, 95% CI = [0.01, 0.38]; *t*_98_ = 2.06, *p* = 0.042; figure 4*b*), indicating that the same acoustic parameters that have been linked to discrimination of other voices [18] account for VD of the self-voice. Neither sound conduction (air, bone) nor the type of other-voice (familiar, unfamiliar) affected the relationship between task performance and self-other distance (electronic supplementary material). Further analyses related to acoustic properties—gender differences, alternative voice-space constructions, as well as separate contributions of source (larynx) and filter (vocal tract)—are reported in the electronic supplementary material.

Considering that previous work constructed the voice space by using three vowels (/a/, /i/, /u/) that reflect the extremes of the vowel space area [75], whereas we used only /a/, we repeated analysis with an alternative construction of the second voice-space dimension, which is better tailored for the vowel /a/. The results did not change significantly and are reported in the electronic supplementary material.

Acoustic parameters of all participants’ voices from all studies are also reported in the electronic supplementary material.

### 3.5. Control self-voice recognition task

Findings from Studies 1–3 indicate that bone conduction facilitates self-other VD, regardless of other-voice familiarity. However, those findings could be misleading if participants are unable to recognize their voice without voice morphing. Moreover, it remains unclear whether the bone conduction advantage generalizes to other self-voice tasks, similar to the ones reported in previous work, where no voice morphing was used. Thus, at the end of Study 3, participants performed a control self-voice recognition task in which all three unmorphed voices were used as stimuli within the same experimental block.

**Figure 5.**
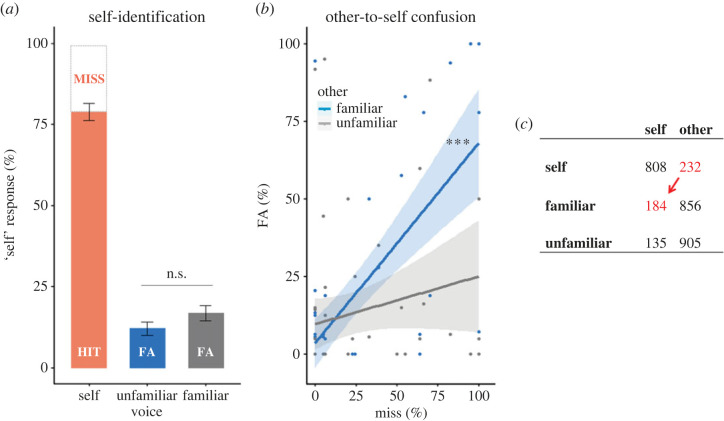
Study 3: control task. The bar plot (*a*) indicates mean rates of ‘self’ responses occurring for each type of voice stimuli—hit rate for self- and false-alarm (FA) rates for familiar and unfamiliar voices—whereas the regression plots (*b*) indicate relationships between FA rates for familiar and unfamiliar voice with the miss rate for self-voice. Bar plot whiskers and shaded areas around linear regressions indicate 95% confidence intervals. Although the absolute rate at which familiar and unfamiliar voices was misperceived as self-voice did not differ (*a*), only the familiar-voice misperception was related to self-voice (*b*). In a confusion matrix (*c*), that maps three vocal stimuli (self, familiar, unfamiliar; rows) to participants' responses ('self', 'other'; columns), this could be represented as a shift of falsely labelled self-voice trials mostly towards familiar voice (red arrow). *** *p* < 0.001.

Mixed-effects binomial regressions showed that there were more ‘self’ responses in self-voice trials (i.e. hit rate, mean = 0.79, 95% CI = [0.72, 0.85]) both compared with familiar-voice (i.e. familiar-FA rate, 0.17, [0.11, 0.23]; estimate = −3.23, *Z* = −17.48, *p* < 0.001) and unfamiliar-voice trials (i.e. unfamiliar-FA rate, 0.13, [0.08, 0.18]; estimate = −3.44, *Z* = −17.93, *p* < 0.001) (figure 5*a*). There were no differences in ‘self’ responses between trials with familiar and unfamiliar voices (i.e. FA rates, estimate = −0.21, *Z* = −1.1, *p* = 0.273). The main effect of Conduction was not significant (estimate = 0, *Z* = 0.03, *p* = 0.98) nor was there a Conduction by Voice interaction (estimate = −0.13, *Z* = −0.46, *p* = 0.643). These data show that, although participants were mostly correct in identifying their own unmorphed voice (79% hit rate), they also misinterpreted both familiar and unfamiliar other voices as their own during some trials (17% and 13% FAs), indicating that recognizing own voice in a recording even without additional transformations (e.g. morphing with other voices) of is not as trivial as it might seem. Thus, nine out of 52 participants who could not recognize their voice in more than half of the self-voice trials (i.e. accuracy lower than 50%) in the control task were considered as outliers and excluded from the analysis in the discrimination tasks above.

### 3.6. Other-to-self-voice confusion

The self-voice recognition task indicated that in a non-negligible amount trials participants misperceived either familiar (17%) or unfamiliar (13%) voice as their own (i.e. responded ‘self’ for ‘other’ stimuli—FA), but it remains unknown whether the two FA rates (unfamiliar FA and familiar FA) are related to each other (i.e. indicating a general ownership over other voices), and to a decrease in recognition of their own voice (i.e. to the miss rate—responding ‘other’ to ‘self’ stimuli—indicating that other voices were confused with self-voice, a self-voice disownership). Thus, we ran correlation analyses between the two FA rates and miss rate. A significant correlation between the two FAs would indicate that participants who misperceived one type of voice as self-voice also misperceived another, suggesting a general tendency to misperceive other voices as self-voice, regardless of voice familiarity. A significant correlation between miss rate and the type of FA would indicate that another voice is confused as self-voice.

Pearson's product-moment correlation did not show a significant relationship between the two FA rates (*r* = −0.07, 95% CI = [−0.34, 0.21]; *t*_50_ = −0.49, *p* = 0.624), showing that participants either misperceived the familiar or the unfamiliar voice as their own, but independently. The correlation analysis identified a significant positive relationship (*r* = 0.67, 95% CI = [0.48, 0.79]) between miss rate and familiar-FA rate (*t*_50_ = 6.31, *p* < 0.001), while there was no significant relationship (*t*_50_ = 1.46, *p* = 0.151) between miss rate and unfamiliar-FA rate (*r* = 0.2, 95% CI = [−0.07, 0.45]) (figure 5*b*). Pearson and Filon's *z*-test identified a stronger relationship between miss and familiar-FA compared with unfamiliar-FA rates (*z* = 2.86, *p* = 0.004), indicating that participants were confusing self-voice more with the familiar compared with the unfamiliar voice. This shows that, although familiar-to-self-voice FA rate did not occur significantly more than unfamiliar-to-self FA rate (figure 5*a*), only familiar-to-self FA rate was related to the miss rate of self-voice (figure 5*b*), indicating that (only) familiar-other voice is confused as self-voice. This can also be illustrated through a confusion matrix (figure 5*c*), by suggesting that falsely identified self-voice trials mostly shifted towards familiar, and not towards unfamiliar voice (red arrow on figure 5*c*). This is because the participants who answered ‘self’ for the familiar voice did not answer ‘self’ for the actual self-voice, while participants who answered ‘self’ for the unfamiliar voice also answered ‘self’ for the actual self-voice. The first category of participants thus confused familiar-voice as self-voice, whereas the second category probably had a bias of answering ‘self’. No correlations were affected by sound conduction type (electronic supplementary material).

Collectively, these results suggest that we are prone to confusing a familiar voice as our own, seemingly because it is familiar, and regardless of the acoustic similarity between the two voices. This sheds new light on the effects of familiarity present in stimuli associated with the self.

## 4. Discussion

As our main means of verbal communication, our voice is an integral part of our identity and our self. Although traditionally thought of as a purely auditory signal, self-voice is a multi-modal percept that also involves vibrotactile input [32,33], at least when we are actively speaking. Perceiving our voice passively, that is, presented through air-conduction loudspeakers, differs in two ways: (i) it sounds different, since it is not (low-pass) filtered as a result of passing through the skull and (ii) it lacks multi-modal input resulting from speech production. Conversely, this leads to a reduced ability to recognize ourselves in air-conducted recordings [16,36,37,41–43]. This has significantly impeded self-voice-related research, rendering it one of the least investigated aspects of self-consciousness [1]. While previous work has tried approximating the natural self-voice by applying acoustic transformations [50–54], we here focused on the multi-modal aspect of the self-voice by presenting stimuli through a commercially available bone conduction headset. This allowed us to pinpoint perceptual specificities related to self-voice, ranging from low-level acoustic to high-level cognitive aspects such as familiarity and previous exposure.

### 4.1. Vibrotactile stimulation

Studies 1 and 3, demonstrated that self-other VD improves with bone compared with air conduction. As we argue below, this demonstrates the importance of vibrotactile signals generated by the bone conduction vibrations in addition to its low-frequency filtering of the auditory signal or voice familiarity.

Acoustic transformations resulting from bone conduction play an important role in self-voice perception. They might constitute an internal model of what the self-voice should sound like, and thus hearing our voice through bone as opposed to air conduction might better approximate this model, resulting in higher performance in the self-other VD task. However, acoustic transformations are difficult to manipulate experimentally, as the exact transfer function of the skull and other head tissues has not yet been formally defined and remains a topic of ongoing research in acoustics [31,76]. Studies that tried to experimentally alter the sound of the air-conducted self-voice in order to render it more similar to the bone-conducted one yielded contradictory and sometimes inconsistent results [50–54]. We therefore opted for presenting self-voice stimuli directly through bone conduction. This results in both acoustic transformations of the sound of our voice and in accompanying vibrotactile stimulation, which has been neglected in previous studies.

Importantly, the bone conduction effect was specific to the involvement of the self-voice, and not for other familiar voices, as bone conduction did not improve familiar-other VD (Studies 2 and 3). This separates the effect of bone conduction from the effect of voice familiarity: if bone conduction effects were related to neural mechanisms associated with familiarity, then similar differences should have occurred in the familiar-other task. As this was not the case, it is likely that the bone conduction effects are specific to self-voice, not because self-voice is a stimulus we are familiar with but because it involves a dedicated neural system associated with the self. Moreover, a lack of perceptual differences between air and bone conduction in the familiar-other task further supports the importance of vibrotactile stimulation accompanying bone conduction, as opposed to physical transformations to self-voice stimuli (e.g. deeper sound due to filter properties of bone tissue). Namely, if only physical transformations were to account for the bone conduction effect in the self-other task, the opposite effect should be observed in the familiar-other task (i.e. a disadvantage of bone conduction for familiar voices), given that we are not used to hearing familiar voices transformed in such a way (e.g. deeper voices of acquaintances). As equivalent acoustic transformations applied by the same bone conduction headset led to perceptual differences for self-voice and not familiar-voice stimuli, we suggest that concomitant vibrotactile signals, usually exclusive to one's own voice, play an important role in self-voice processing. This suggests that self-voice is a multi-modal construct and consolidates evidence in favour of self-related processes in the perception of voices as it has been shown for the perception of faces [14,77].

### 4.2. Previous exposure

Our results further demonstrate better performance in self-voice tasks in people with higher previous exposure to self-voice via recordings as has previously been demonstrated, e.g. in radio announcers [34]. By targeting the effect of previous exposure of self-voice in a more controlled way—in half of experimental blocks of Study 1—the unmorphed self-voice was shown to participants before the self-other VD task. This was done in the second half of the experiment so as not to affect the performance in the first half of experiment. Confirming previous findings [34,35], we found that prior exposure facilitated self-other VD. Participants' performance improved when they heard their unmorphed voice recording immediately before the task: by hearing an unmorphed recording of their voice prior to task execution, participants could have created an arbitrary strategy of recognizing that specific voice recording in a voice morph, regardless of whether they associated the recording with themselves or not. For instance, when hearing two unmorphed voices before the task, participants could focus on one acoustic property (e.g. a higher pitch in one of the recordings) and use that property as a reference against which voice morphs are compared. Without previous exposure, however, there was no pre-exposure-based additional reference that participants could rely on to complete the task, and they had to rely on their internal self-voice representation. The fact that there was no effect of previous exposure on the familiar-other task in Study 2 also suggests that the effect of previous exposure in Study 1 did not occur because it was manipulated only in the second half of the experiment, controlling for task habituation effects.

The addition or omission of pre-exposure to the own voice is also important for understanding the contribution of bone conduction in Study 1. Bone conduction only improved self-other VD when this discrimination was based on an internal representation of the own voice as opposed to comparing it with the pre-exposure stimulus, which essentially rendered the task easier. This is supported by the familiar-other VD findings (Study 2), which showed that bone conduction did not affect performance in blocks with familiar versus unfamiliar voices (with and without previous exposure). This suggests that bone conduction facilitation is only found for the self-voice, but not for familiar voices that are mainly based on auditory cues.

### 4.3. Familiarity and acoustic parameters

We performed additional analyses demonstrating that both familiarity processing and acoustic differences contribute to self-other VD. On the one hand, the results of our correlation analysis between miss and FA rates in the control self-voice recognition task show that self-voice perception inevitably involves some familiarity processing. Thus, a failure to recognize own voice (miss rate) was correlated with familiar-to-self, and not with unfamiliar-to-self-voice misattribution (FAs), regardless of acoustic similarity between the three voices. This suggests that self-voice was more confused with a familiar than with an unfamiliar other voice and that familiarity mechanisms [56,57] also bias self-voice perception. An analogy in the visual domain would be observing that participants confuse their own face more with a familiar as opposed to an unfamiliar other face, despite unfamiliar face being physically more similar (e.g. based on eye colour or nose shape). On the other hand, our voice-space analysis indicates that, to a certain extent, also low-level acoustic properties have an impact on self-voice recognition (for detailed results and discussion, see electronic supplementary material). Without ‘*a priori*’ hypotheses, we placed our participants' voices in other-centred voice spaces [18] and observed a correlation between acoustic distances and discriminability ratings. This supports the involvement of a third factor, low-level acoustic processes, in self-other VD and shows that the acoustic differences accounting for discrimination of other voices extend to self-voice, that should be further explored (see electronic supplementary material). In sum, these findings show that both familiarity mechanisms and acoustic processes contribute to self-voice perception, and future studies should identify ways to delineate the corresponding contributions of these factors.

### 4.4. Task sensitivity

While previous self-voice tasks have been characterized by ceiling effects, the paradigm proposed here is able to capture inter-subject variability, which allows us to dissect perceptual specificities (e.g. a bias, general sensitivity, or effects specific to self- or other-voice perception) and personalize studies of self-other VD. Most participants in Studies 1 and 3 (as well as in our follow-up EEG study [59]) spontaneously reported that they perceived the self-other VD task to be very difficult and showed poor metacognition; that is, they misjudged their ability of successfully performing the task. Moreover, in Study 1, we observed large differences in performance across participants. That is why, in Study 3, in addition to increasing the sample size based on the power analysis of Study 1 (see Methods), we introduced a control task at the end of experiment, to narrow down the self-other VD analysis to include only those participants able to recognize their own unmorphed voice. To our surprise, nine out of 52 (17.3%) participants could not recognize their unmorphed voice in more than half of self-recognition task's trials, and were thus excluded from the analysis in the VD tasks. This shows that recognizing the own voice in short vocalizations (e.g. phoneme /a/ lasting for 500 ms) is not as trivial as it might seem (even without voice morphing), although it is shown to suffice for speaker identification [78]. Moreover, a decrease in difficulty between the control self-voice recognition task and self-other VD task might account for a lack of differences between air and bone conduction in the control task. Namely, it is possible that the bone conduction advantages for self-voice perception are detectable only in tasks that are sensitive enough to detect them, which was probably not the case for the control task not involving voice morphing. The bone conduction advantage also disappeared in the blocks with previous exposure in Study 1. As indicated above, previous exposure enabled participants to have other strategies to perform self-other VD task, which probably made the task easier. This could have led to similar ceiling effects in which the bone conduction advantages are not perceivable. Importantly, a change in bone conduction effects was not observed in Study 2, which manipulated previous exposure in the same way as Study 1, but for familiar–unfamiliar VD. This suggests that task difficulty matters for the bone conduction effects, but only when tasks involve self-voice.

### 4.5. Other-dominant voice morphs

Although bone conduction improved the performance in self-other VD both in Studies 1 and 3 (a steeper slope of the psychometric curve), the specific morphs for which the difference in performance was biggest differ between the studies (self-dominant morphs for Study 1, figure 2*a*; other-dominant for Study 3, figure 3*a*). We believe that this difference is mainly due to (i) a smaller sample size in Study 1 (N1 = 16, N3 = 43) and (ii) a poorer sound quality due to a different air-conducting medium (laptop loudspeakers in Study 1 as compared with headphones in Study 3). We believe that the bone conduction effect is indeed specific for other-dominant self-other voice morphs, as it occurred in Study 3 for two different tasks (self-other and self-familiar), and, importantly, it was replicated in a follow-up EEG study with an independent cohort of participants performing the same self-other task with five times more trials [59]. This suggests that, rather than labelling an ambiguous voice as ‘self’, bone conduction facilitates discarding an ambiguous voice as being ‘not self’. In other words, our data show that bone conduction specifically facilitates making a ‘not self’ judgement in scenarios of vocal ambiguity.

### 4.6. Motor signals

It is important to note that natural self-voice perception also involves motor signals related to speech production, that were not tested here. The presence of such motor signals and the associated intraoral and pharyngeal sensory speech-related cues may also exert additional effects on self-other VD, as opposed to familiar-other VD. An equivalent study in which participants would hear self-other voice morphs triggered by vocalization onset could investigate the potential role of motor signals, in addition to or as opposed to vibrotactile effects. This would, however, be challenging to investigate, as speech-related motor signals and the resulting vibrotactile sensations cannot be experimentally separated during natural speech.

### 4.7. Headset frequency response

The observed bone conduction effects might partially be accounted for by the differences in frequency responses between bone- and air-conduction headsets. Namely, it is possible that our bone conduction headset has a low-frequency emphasis that renders self-voice more familiar to the listeners, thereby increasing the self-other VD performance. To verify that account, we would have to measure and compare the frequency responses of our headsets. However, Manning *et al*. [79] measured the frequency response of the exact same bone conduction headset used in our studies and observed it to be quite flat, even for the higher frequencies. By contrast, the response of the air-conduction headset in this latter study had a marked low-frequency emphasis, that could be expected for a bone conduction headset. Importantly, however, even if the sound from both headsets had the same frequency response, the sound that enters the inner ears coming from these two sources (outer ears for air-conduction headset and cheekbones for bone conduction) will always differ in frequency response, with the bone-conducted sound being filtered by the skull and other tissues in the head. As the exact transfer function of the head is still unknown [31,76], and previous attempts of filtering the air-conducted sound did not yield conclusive results [50–54], it remains difficult to isolate the contribution of acoustic filtering in present effects, especially with respect to the contribution of concomitant vibrotactile stimulation.

### 4.8. Impact and clinical relevance

The impact of this work is threefold. First, by shifting the classical perspective on self-voice away from purely auditory to multi-modal, these findings incorporate self-voice into multi-sensory accounts of self-consciousness [80–83]. According to these accounts, the sense of self is fundamentally based on the continuous integration of multi-sensory bodily signals, including tactile, proprioceptive, interoceptive, visual, and auditory signals [10,81,84–86]^.^ Correspondingly, we show that integration of auditory and vibrotactile signals increases the recognition of our voice, that is an integral part of our self. Second, by introducing a method which improves auditory self-identification, we propose a new approach to addressing self-voice-related research questions. Based on these findings, future studies can avoid presenting self-voice stimuli through traditional air-conducting media, especially considering the increasing availability of bone conduction headsets. Finally, this work could serve as a scaffold for clinical investigations of a very common [23,24] and highly distressing [25,26] psychiatric symptom—AVH, i.e. ‘hearing voices’—as they have been proposed to arise as a self-other VD deficit [19,22,87–89]. Specifically, characterizing differences in self-other VD curves in voice-hearers compared with controls (e.g. quantifying a bias to hear other-voice and relating it to clinical measures) could deepen the understanding as well as challenge this prominent account for AVH aetiology. Collectively, our findings demonstrate the importance of bone conduction with respect to self-voice perception and shed new light on the phenomenology of the self by portraying self-voice as a fundamentally multi-modal composition in which both familiarity and acoustic properties play a significant role.

## Data Availability

The datasets generated and/or analysed during the current study are available in the open science framework (OSF) repository: https://osf.io/uxvh7/. The data are provided in the electronic supplementary material [90].

## References

[1] Holzman PS, Rousey C. 1966 The voice as a percept. J. Pers. Soc. Psychol. **4**, 79-86. (10.1037/h0023518) 5965194

[2] Holzman PS, Rousey C, Snyder C. 1966 On listening to one's own voice: effects on psychophysiological responses and free associations. J. Pers. Soc. Psychol. **4**, 432-441. (10.1037/h0023790) 5969999

[3] Lee M, Drinnan M, Carding P. 2005 The reliability and validity of patient self-rating of their own voice quality. Clin. Otolaryngol. **30**, 357-361. (10.1111/J.1365-2273.2005.01022.X) 16209679

[4] Daryadar M, Raghibi M. 2015 The effect of listening to recordings of one's voice on attentional bias and auditory verbal learning. Int. J. Psychol. Stud. **7**, 155. (10.5539/ijps.v7n2p155)

[5] Holzman PS, Berger A, Rousey C. 1967 Voice confrontation: a bilingual study. J. Pers. Soc. Psychol. **7**, 423-428. (10.1037/h0025233) 6065872

[6] Qin P, Wang M, Northoff G. 2020 Linking bodily, environmental and mental states in the self—a three-level model based on a meta-analysis. Neurosci. Biobehav. Rev. **115**, 77-95. (10.1016/j.neubiorev.2020.05.004) 32492474

[7] Northoff G, Heinzel A, de Greck M, Bermpohl F, Dobrowolny H, Panksepp J. 2006 Self-referential processing in our brain—a meta-analysis of imaging studies on the self. Neuroimage **31**, 440-457. (10.1016/j.neuroimage.2005.12.002) 16466680

[8] Sui J, Humphreys GW. 2017 The ubiquitous self: what the properties of self-bias tell us about the self. Ann. N Y Acad. Sci. **1396**, 222-235. (10.1111/nyas.13197) 27918835 PMC6029667

[9] Kircher T, David AS. 2003 Self-consciousness: an integrative approach from philosophy, psychopathology and the neurosciences. In The self in neuroscience and psychiatry (eds T Kircher, AS David), pp. 445-473. Cambridge, UK: Cambridge University Press.

[10] Blanke O, Slater M, Serino A. 2015 Behavioral, neural, and computational principles of bodily self-consciousness. Neuron **88**, 145-166. (10.1016/j.neuron.2015.09.029) 26447578

[11] Uddin LQ, Kaplan JT, Molnar-Szakacs I, Zaidel E, Iacoboni M. 2005 Self-face recognition activates a frontoparietal ‘mirror’ network in the right hemisphere: an event-related fMRI study. Neuroimage **25**, 926-935. (10.1016/j.neuroimage.2004.12.018) 15808992

[12] Frassinetti F, Maini M, Romualdi S, Galante E, Avanzi S. 2008 Is it mine? Hemispheric asymmetries in corporeal self-recognition. J. Cogn. Neurosci. **20**, 1507-1516. (10.1162/jocn.2008.20067) 18211238

[13] Ma Y, Han S. 2010 Why we respond faster to the self than to others? An implicit positive association theory of self-advantage during implicit face recognition. J. Exp. Psychol. Hum. Percept. Perform. **36**, 619. (10.1037/a0015797) 20515192

[14] Platek SM, Wathne K, Tierney NG, Thomson JW. 2008 Neural correlates of self-face recognition: an effect-location meta-analysis. Brain Res. **1232**, 173-184. (10.1016/J.BRAINRES.2008.07.010) 18656465

[15] Nakamura K et al. 2001 Neural substrates for recognition of familiar voices: a PET study. Neuropsychologia **39**, 1047-1054. (10.1016/S0028-3932(01)00037-9) 11440757

[16] Allen PP, Amaro E, Fu CHY, Williams SCR, Brammer M, Johns LC, McGuire PK. 2005 Neural correlates of the misattribution of self-generated speech. Hum. Brain Mapp. **26**, 44-53. (10.1002/hbm.20120) 15884023 PMC6871759

[17] Kaplan JT, Aziz-Zadeh L, Uddin LQ, Iacoboni M. 2008 The self across the senses: an fMRI study of self-face and self-voice recognition. Soc. Cogn. Affect. Neurosci. **3**, 218-223. (10.1093/scan/nsn014) 19015113 PMC2566765

[18] Baumann O, Belin P. 2010 Perceptual scaling of voice identity: common dimensions for different vowels and speakers. Psychol. Res. **74**, 110-120. (10.1007/s00426-008-0185-z) 19034504

[19] Frith CD, Done DJ. 1989 Experiences of alien control in schizophrenia reflect a disorder in the central monitoring of action. Psychol. Med. **19**, 359-363. (10.4324/9781315630502) 2762440

[20] Frith CD. 1992 The cognitive neuropsychology of schizophrenia. Hillsdale, NJ: Lawrence Erlbaum Associates, Inc.

[21] Allen P, Aleman A, McGuire PK. 2007 Inner speech models of auditory verbal hallucinations: evidence from behavioural and neuroimaging studies. Int. Rev. Psychiatry **19**, 407-415. (10.1080/09540260701486498) 17671873

[22] Ford JM, Mathalon DH. 2005 Corollary discharge dysfunction in schizophrenia: can it explain auditory hallucinations? Int. J. Psychophysiol. **58**, 179-189. (10.1016/j.ijpsycho.2005.01.014) 16137779

[23] Bauer SM et al. 2011 Culture and the prevalence of hallucinations in schizophrenia. Compr. Psychiatry **52**, 319-325. (10.1016/j.comppsych.2010.06.008) 21497227

[24] Nayani TH, David AS. 1996 The auditory hallucination: a phenomenological survey. Psychol. Med. **26**, 177-189. (10.1017/s003329170003381x) 8643757

[25] Harkavy-Friedman JM, Kimhy D, Nelson EA, Venarde DF, Malaspina D, Mann JJ. 2003 Suicide attempts in schizophrenia: the role of command auditory hallucinations for suicide. J. Clin. Psychiatry **64**, 871-874. (10.4088/JCP.v64n0803) 12927000

[26] Kelleher I, Lynch F, Harley M, Molloy C, Roddy S, Fitzpatrick C, Cannon M. 2012 Psychotic symptoms in adolescence index risk for suicidal behavior: findings from 2 population-based case-control clinical interview studies. Arch. Gen. Psychiatry **69**, 1277-1283. (10.1001/archgenpsychiatry.2012.164) 23108974

[27] Békésy GV. 1949 The structure of the middle ear and the hearing of one's own voice by bone conduction. J. Acoust. Soc. Am. **21**, 217-232. (10.1121/1.1906501)

[28] Reinfeldt S, Östli P, Håkansson B, Stenfelt S. 2010 Hearing one's own voice during phoneme vocalization—transmission by air and bone conduction. J. Acoust. Soc. Am. **128**, 751-762. (10.1121/1.3458855) 20707445

[29] Tonndorf J. 1976 Bone conduction. In Auditory system: handbook of sensory physiology (eds WD Keidel, WD Neff), pp. 37-84. Berlin, Germany: Springer.

[30] Wheatstone C. 1827 Experiments on audition. Q. J. Sci. Lit. Art **24**, 67-72.

[31] Stenfelt S. 2011 Acoustic and physiologic aspects of bone conduction hearing. Advances in Oto-Rhino-Laryngology **71**, 10-21. (10.1159/000323574) 21389700

[32] Tremblay S, Shiller DM, Ostry DJ. 2003 Somatosensory basis of speech production. Nature **423**, 866-869. (10.1038/nature01710) 12815431

[33] Ito T, Tiede M, Ostry DJ. 2009 Somatosensory function in speech perception. Proc. Natl Acad. Sci. USA **106**, 1245-1248. (10.1073/pnas.0810063106) 19164569 PMC2633542

[34] Rousey C, Holzman PS. 1967 Recognition of one's own voice. J. Pers. Soc. Psychol. **6**, 464-466. (10.1037/h0024837) 6082480

[35] Olivos G. 1967 Response delay, psychophysiologic activation, and recognition of one's own voice. Psychosom. Med. **29**, 433-440. (10.1097/00006842-196709000-00003) 6059916

[36] Shuster LI. 1998 The perception of correctly and incorrectly produced /r/. J. Speech Lang. Hear. Res. **41**, 941-950. (10.1044/jslhr.4104.941) 9712139

[37] Rosa C, Lassonde M, Pinard C, Keenan JP, Belin P. 2008 Investigations of hemispheric specialization of self-voice recognition. Brain Cogn. **68**, 204-214. (10.1016/j.bandc.2008.04.007) 18541355

[38] Allen PP, Johns LC, Fu CHY, Broome MR, Vythelingum GN, McGuire PK. 2004 Misattribution of external speech in patients with hallucinations and delusions. Schizophr. Res. **69**, 277-287. (10.1016/j.schres.2003.09.008) 15469199

[39] Candini M, Avanzi S, Cantagallo A, Zangoli MG, Benassi M, Querzani P, Lotti EM, Iachini T, Frassinetti F. 2018 The lost ability to distinguish between self and other voice following a brain lesion. Neuroimage Clin. **18**, 903-911. (10.1016/j.nicl.2018.03.021) 29876275 PMC5988014

[40] Candini M, Zamagni E, Nuzzo A, Ruotolo F, Iachini T, Frassinetti F. 2014 Who is speaking? Implicit and explicit self and other voice recognition. Brain Cogn. **92**, 112-117. (10.1016/j.bandc.2014.10.001) 25463145

[41] Hughes SM, Nicholson SE. 2010 The processing of auditory and visual recognition of self-stimuli. Conscious Cogn. **19**, 1124-1134. (10.1016/j.concog.2010.03.001) 20347341

[42] Schuerman WL, Meyer A, McQueen JM. 2015 Do we perceive others better than ourselves? A perceptual benefit for noise-vocoded speech produced by an average speaker. PLoS ONE **10**, 1-18. (10.1371/journal.pone.0129731) PMC4489924 26134279

[43] Gur RC, Sackeim HA. 1979 Self-deception: a concept in search of a phenomenon. J. Pers. Soc. Psychol. **37**, 147-169. (10.1037/0022-3514.37.2.147)

[44] Douglas W, Gibbins K. 1983 Inadequacy of voice recognition as a demonstration of self-deception. J. Pers. Soc. Psychol. **44**, 589-592. (10.1037/0022-3514.44.3.589) 6834242

[45] Zhou. A, Yanbing H, Lu X, Shen S, Chen X, Pan T. 2019 Investigations of verbal cues and self-voice perception model. In Proc. of the Int. Joint Conf. on Information, Media, and Engineering, IJCIME 2019, Osaka, Japan, 17 December, pp. 468-471. IEEE.

[46] Conde T, Gonçalves ÓF, Pinheiro AP. 2018 Stimulus complexity matters when you hear your own voice: attention effects on self-generated voice processing. Int. J. Psychophysiol. **133**, 66-78. (10.1016/j.ijpsycho.2018.08.007) 30114437

[47] Pinheiro AP, Rezaii N, Rauber A, Nestor PG, Spencer KM, Niznikiewicz M. 2017 Emotional self–other voice processing in schizophrenia and its relationship with hallucinations: ERP evidence. Psychophysiology **54**, 1252-1265. (10.1111/psyp.12880) 28474363

[48] Conde T, Gonçalves ÓF, Pinheiro AP. 2016 The effects of stimulus complexity on the preattentive processing of self-generated and nonself voices: an ERP study. Cogn. Affect. Behav. Neurosci. **16**, 106-123. (10.3758/s13415-015-0376-1) 26415897

[49] Conde T, Gonçalves ÓF, Pinheiro AP. 2015 Paying attention to my voice or yours: an ERP study with words. Biol. Psychol. **111**, 40-52. (10.1016/j.biopsycho.2015.07.014) 26234962

[50] Vurma A. 2014 The timbre of the voice as perceived by the singer him-/herself. Logoped. Phoniatr. Vocol. **39**, 1-10. (10.3109/14015439.2013.775334) 23510260

[51] Won SY, Berger J, Slaney M. 2014 Simulation of one’s own voice in a two-parameter model. In Proc. of the Int. Conf. on Music Perception and Cognition (ICMPC)*,* Seoul, South Korea*,* 4 August. College of Music, Yonsei University.

[52] Shuster LI, Durrant JD. 2003 Toward a better understanding of the perception of self-produced speech. J. Commun. Disord. **36**, 1-11. (10.1016/S0021-9924(02)00132-6) 12493635

[53] Kimura M, Yotsumoto Y. 2018 Auditory traits of ‘own voice’. PLoS ONE **13**, 1-16. (10.1371/journal.pone.0199443) PMC6019673 29944698

[54] Maurer D, Landis T. 1990 Role of bone conduction in the self-perception of speech. Folia Phoniatr. Logop. **42**, 226-229. (10.1159/000266070) 2283129

[55] Kawahara H, Morise M, Banno H, Skuk VG. 2013 Temporally variable multi-aspect N-way morphing based on interference-free speech representations. In Asia-Pacific Signal and Information Processing Association Annual Summit and Conf., APSIPA 2013, pp. 1-10. IEEE.

[56] Sidtis D, Kreiman J. 2011 In the beginning was the familiar voice: personally familiar voices in the evolutionary and contemporary biology of communication. Integr. Psychol. Behav. Sci. **46**, 146-159. (10.1007/S12124-011-9177-4) 21710374 PMC3224673

[57] Stevenage SV. 2018 Drawing a distinction between familiar and unfamiliar voice processing: a review of neuropsychological, clinical and empirical findings. Neuropsychologia **116**, 162-178. (10.1016/j.neuropsychologia.2017.07.005) 28694095

[58] Orepic P, Rognini G, Kannape OA, Faivre N, Blanke O. 2021 Sensorimotor conflicts induce somatic passivity and louden quiet voices in healthy listeners. Schizophr. Res. **231**, 14. (10.1016/j.schres.2021.03.014) 33866262

[59] Iannotti GR, Orepic P, Brunet D, Koenig T, Alcoba-Banqueri S, Garin DFA, Schaller K, Blanke O, Michel CM. 2021 EEG spatiotemporal patterns underlying self-other voice discrimination. Cereb. Cortex **32**, 1978-1992. (10.1093/CERCOR/BHAB329) PMC9070353 34649280

[60] Kleiner M, Brainard DH, Pelli DG, Ingling A, Murray R, Broussard A, Ingling R, Murray C. 2007 What's new in Psychtoolbox-3? Perception **36**, 1-16. (10.1068/v070821)

[61] R Core Team. 2020 *R: a language and environment for statistical computing*. Vienna, Austria: R Foundation for Statistical Computing. See https://www.r-project.org/.

[62] Bates D, Mächler M, Bolker BM, Walker SC. 2015 Fitting linear mixed-effects models using lme4. J. Stat. Softw. **67**, 1-48. (10.18637/jss.v067.i01)

[63] Kuznetsova A, Brockhoff PB, Christensen RHB. 2018 lmerTest package: tests in linear mixed effects models. J. Stat. Softw. **82**, 1-26. (10.18637/jss.v082.i13)

[64] Diedenhofen B, Musch J. 2015 Cocor: a comprehensive solution for the statistical comparison of correlations. PLoS ONE **10**, 1-12. (10.1371/journal.pone.0121945) PMC4383486 25835001

[65] Lüdecke D. 2018 sjPlot: data visualization for statistics in social science. R package version 2.6.2. See https://cran.r-project.org/package=sjPlot.

[66] Wickham H. 2016 Ggplot2: elegant graphics for data analysis. New York: NY: Springer-Verlag.

[67] Green P, Macleod CJ. 2016 SIMR: an R package for power analysis of generalized linear mixed models by simulation. Methods Ecol. Evol. **7**, 493-498. (10.1111/2041-210X.12504)

[68] Bürkner PC. 2017 brms: an R package for Bayesian multilevel models using Stan. J. Stat. Softw. **80**, 1-28. (10.18637/jss.v080.i01)

[69] Pearson K, Filon LNG. 1898 Mathematical contributions to theory of evolution: IV. On the probable errors of frequency constants and on the influence of random selection and correlation. Proc. R. Soc. Lond. **62**, 229-311. (10.1098/rsta.1898.0007)

[70] Latinus M, McAleer P, Bestelmeyer PEG, Belin P. 2013 Norm-based coding of voice identity in human auditory cortex. Curr. Biol. **23**, 1075-1080. (10.1016/j.cub.2013.04.055) 23707425 PMC3690478

[71] López S, Riera P, Assaneo MF, Eguía M, Sigman M, Trevisan MA. 2013 Vocal caricatures reveal signatures of speaker identity. Sci. Rep. **3**, 1-7. (10.1038/srep03407) PMC3847701 24297083

[72] Skuk VG, Schweinberger SR. 2013 Gender differences in familiar voice identification. Hear. Res. **296**, 131-140. (10.1016/j.heares.2012.11.004) 23168357

[73] Ghazanfar AA, Rendall D. 2008 Evolution of human vocal production. Curr. Biol. **18**, R457-R460. (10.1002/jcu.1870190508) 18522811

[74] Boersma P, Weenink D. 2020 Praat: doing phonetics by computer [Computer program]. Version 6.1.12. See http://www.praat.org/ (accessed on 13 April 2020).

[75] Berisha V, Sandoval S, Utianski R, Liss J, Spanias A. 2014 Characterizing the distribution of the quadrilateral vowel space area. J. Acoust. Soc. Am. **135**, 421. (10.1121/1.4829528) 24437782 PMC3985878

[76] Stenfelt S. 2016 Model predictions for bone conduction perception in the human. Hear. Res. **340**, 135-143. (10.1016/j.heares.2015.10.014) 26657096

[77] Alzueta E, Melcón M, Poch C, Capilla A. 2019 Is your own face more than a highly familiar face? Biol. Psychol. **142**, 100-107. (10.1016/j.biopsycho.2019.01.018) 30738092

[78] Zarate JM, Tian X, Woods KJP, Poeppel D. 2015 Multiple levels of linguistic and paralinguistic features contribute to voice recognition. Sci. Rep. **5**, 11475. (10.1038/srep11475) 26088739 PMC4473599

[79] Manning C, Mermagen T, Scharine A. 2017 The effect of sensorineural hearing loss and tinnitus on speech recognition over air and bone conduction military communications headsets. Hear. Res. **349**, 67-75. (10.1016/j.heares.2016.10.019) 27989949

[80] Blanke O. 2012 Multisensory brain mechanisms of bodily self-consciousness. Nat. Rev. Neurosci. **13**, 556-571. (10.1038/nrn3292) 22805909

[81] Park HD, Blanke O. 2019 Coupling inner and outer body for self-consciousness. Trends Cogn. Sci. **23**, 377-388. (10.1016/j.tics.2019.02.002) 30826212

[82] Tsakiris M. 2017 The multisensory basis of the self: from body to identity to others. Q. J. Exp. Psychol. **70**, 597-609. (10.1080/17470218.2016.1181768) PMC5214748 27100132

[83] Petkova VI, Björnsdotter M, Gentile G, Jonsson T, Li TQ, Ehrsson HH. 2011 From part- to whole-body ownership in the multisensory brain. Curr. Biol. **21**, 1118-1122. (10.1016/j.cub.2011.05.022) 21683596

[84] Blanke O, Metzinger T. 2009 Full-body illusions and minimal phenomenal selfhood. Trends Cogn. Sci. **13**, 7-13. (10.1016/j.tics.2008.10.003) 19058991

[85] Gallagher S. 2000 Philosophical conceptions of the self: implications for cognitive science. Trends Cogn. Sci. **4**, 14-21. (10.1016/S1364-6613(99)01417-5) 10637618

[86] Jeannerod M. 2003 The mechanism of self-recognition in humans. Behav. Brain Res. **142**, 1-15. (10.1016/S0166-4328(02)00384-4) 12798261

[87] Blakemore SJ, Smith J, Steel R, Johnstone EC, Frith CD. 2000 The perception of self-produced sensory stimuli in patients with auditory hallucinations and passivity experiences: evidence for a breakdown in self-monitoring. Psychol. Med. **30**, 1131-1139. (10.1017/S0033291799002676) 12027049

[88] Moseley P, Fernyhough C, Ellison A. 2013 Auditory verbal hallucinations as atypical inner speech monitoring, and the potential of neurostimulation as a treatment option. Neurosci. Biobehav. Rev. **37**, 2794-2805. (10.1016/j.neubiorev.2013.10.001) 24125858 PMC3870271

[89] Whitford TJ. 2019 Speaking-induced suppression of the auditory cortex in humans and its relevance to schizophrenia. Biol. Psychiatry Cogn. Neurosci. Neuroimaging **4**, 791-804. (10.1016/j.bpsc.2019.05.011) 31399393

[90] Orepic P, Kannape OA, Faivre N, Blanke O. 2023 Bone conduction facilitates self-other voice discrimination. Figshare. (10.6084/m9.figshare.c.6414070) PMC9929504 36816848

